# Acoustophoretic separation of airborne millimeter-size particles by a Fresnel lens

**DOI:** 10.1038/srep43374

**Published:** 2017-03-02

**Authors:** Ahmet Cicek, Nurettin Korozlu, Olgun Adem Kaya, Bulent Ulug

**Affiliations:** 1 Department of Nanoscience and Nanotechnology, Faculty of Arts and Science, Mehmet Akif Ersoy University, 15030 Burdur/Turkey; 2 Department of Computer Education and Educational Technology, Faculty of Education, Inonu University, 44280 Malatya/Turkey; 3 Department of Physics, Faculty of Science, Akdeniz University, 07058 Antalya/Turkey

## Abstract

We numerically demonstrate acoustophoretic separation of spherical solid particles in air by means of an acoustic Fresnel lens. Beside gravitational and drag forces, freely-falling millimeter-size particles experience large acoustic radiation forces around the focus of the lens, where interplay of forces lead to differentiation of particle trajectories with respect to either size or material properties. Due to the strong acoustic field at the focus, radiation force can divert particles with source intensities significantly smaller than those required for acoustic levitation in a standing field. When the lens is designed to have a focal length of 100 mm at 25 kHz, finite-element method simulations reveal a sharp focus with a full-width at half-maximum of 0.5 wavelenghts and a field enhancement of 18 dB. Through numerical calculation of forces and simulation of particle trajectories, we demonstrate size-based separation of acrylic particles at a source sound pressure level of 153 dB such that particles with diameters larger than 0.5 mm are admitted into the central hole, whereas smaller particles are rejected. Besides, efficient separation of particles with similar acoustic properties such as polyethylene, polystyrene and acrylic particles of the same size is also demonstrated.

Acoustophoresis, i.e. the control of the motion of particles by the acoustic radiation force, has been utilized in contact-free and non-invasive manipulation of particles. The term acoustic radiation pressure was coined by Lord Rayleigh^1^ and radiation force on an incompressible spherical particle in an inviscid fluid was first calculated by L. V. King^2^. It has been widely utilized in microfluidics where radiation force is obtained through exciting surface acoustic waves (SAWs) on piezoelectric substrates^3^,^4^,^5^. Standing SAW acoustophoresis has been successfully applied in separating bioparticles with respect to their size as the acoustic radiation and the counteracting Stokes drag force scale with the third and first power of particle size, respectively^6^,^7^,^8^,^9^,^10^. It has already found many practical applications, e.g. isolating bacteria such as *Escherichia coli*^11^, as well as circulating tumor cells from peripheral blood samples^12^. It is even applied for separation of sub-micron bioparticles such as exosomes as a potential means of early diagnosis of cancer^13^.

Contact-free manipulation of larger-scale massive objects is also necessary in areas such as materials processing, bio-chemistry and pharmaceuticals^14^ where elimination of contamination or friction is required^15^. Unlike the case of microfluidics, acoustic manipulation of massive airborne particles does not require sophisticated fabrication procedures or experimental setup. The most common example of acoustophoresis of airborne millimeter-size particles is acoustic levitation^16^,^17^. Besides, levitation of small living animals, such as insects without affecting their vitality was demonstrated^18^. Such means of manipulating objects is called acoustic tweezers^19^. While a standing ultrasonic field facilitates evenly-distributed trapping positions, a non-resonant system where the reflector and transducer axes are not collinear faciliparticle trapping on a curved path^20^. By placing eight transducers on the edges of an octagon, millimeter-size polystyrene particles are trapped at the nodes of a Bessel-function-shaped field^21^. In addition, negative axial radiation forces to pull particles towards the source of an acoustic Bessel beam are theoretically studied^22^,^23^ and experimentally realized by means of a multi-foci Fresnel lens acting as an axicon^24^. The concept is even applied to levitation of large planar objects^25^. Through introducing a flexible adaptable reflector, an acoustic levtitator can be used to suspend either high-density or high-temperature objects in air^26^.

Acoustic trapping can also be achieved without the involvement of a standing field. For instance, levitation of a large polystyrene ball over three Langevin transducers positioned in a tripod fashion is demonstrated^27^. Alternatively, trapping lipid droplets^28^, as well as polystyrene beads^29^ in water, is achieved by focusing beams through transducers with concave surfaces^28^,^29^,^30^. Extension to airborne particles could facilitate low-power particle manipulation, as acoustic intensity is highly enhanced at the focus.

The second aspect of airborne manipulation is particle transport through a dynamic variation of the acoustic field. Contact-free transport of liquid droplets, or even high-aspect-ratio objects such as a tooth pick, by an array of planar Langevin-type transducers backed by a planar reflector to vary temporal and spatial positions of nodes is demonstrated^14^. Particle transport by node movement can also be achieved through a system of two parallel planar metallic plates where an induced phase difference moves the nodes, and thus particles^31^. In addition, by arranging three curved emitter-reflector pairs on the edges of an hexagon, particle orbital transport or spinning can be actuated by temporally-varying the amplitude of each emitter^32^. Besides, rapid translation of millimeter-size polystyrene particles by controlling the distance between the radiating plate and the reflector in a standing-field configuration is demonstrated^15^.

The ability to distinguish airborne particles with respect to either size or material parameters is also essential in many applications, such as quality control in manufacturing or materials processing. Since the radiation force is size dependent, the node displacement was utilized by Skotis *et al*.^33^ through controlling the relative phases of two opposing speakers so that polystyrene beads of 2 mm and 5 mm diameters are translated at different rates. Li *et al*.^34^ developed an acoustic sieve for sub-millimeter-size particles in water by exciting the *A*_0_ Lamb mode of a brass plate through a phononic crystal. Through the combined action of the resonant ultrasonic field, particles with different sizes can be captured on the surface of the sieve by adjusting the applied power^34^. Similarly, material-based sorting of spherical glass and tin particles is also demonstrated^34^.

Acoustic particle manipulation through focused waves discussed above by means of curved transducer surfaces^28^,^29^,^30^ can alternatively be achieved by flat acoustic lenses in air. Examples of acoustic lensing include negative-refraction^35^ and gradient-index^36^ focusing by two-dimensional (2D) phononic crystals. Acoustic focusing in 2D can also be achieved by optimizing the positions of a finite number of aperiodic scatterers in air^37^,^38^. While these approaches lead to focusing acoustic energy on a plane, fully three-dimensional (3D) lenses should be considered to focus on a point. The idea of aperiodic scatterers is extended to 3D axisymmetric lenses where a finite number of metallic rings are employed^39^. In contrast, acoustic Fresnel lenses offer a more compact means of focusing.

Fresnel lenses are usually obtained by concentric protrusions, grooves or perforations on a thin solid disk^40^,^41^,^42^ where constructive interference of diffracted wave components by adjusting the radii of perturbations^42^. When the disk thickness is half the wavelength, a combination of Fabry-Perot resonances across its thickness and cavity resonances on its surface is responsible from the focusing behavior^41^. A more compact design involves alternately stacked flat and curled channels to introduce a half-wavelength delay through the curled channels^41^. An active planar metasurface Fresnel lens employing concentric piezoelectric PZT-5H rings to obtain adjustable focal length and resolution is also demonstrated^43^. Acoustic Fresnel lenses with perforations can be a good means for acoustophoretic separation of particles, as they may possess central perforations size of which can be designed for the desired separation efficiency.

In this work, we numerically demonstrate tunable separation of airborne millimeter-size solid particles by a low-frequency ultrasonic wave through a Fresnel lens. We show that subwavelength-focused ultrasonic field is capable of separating particles based on either their size or material composition where the lens acts like a sieve. The mechanism is based on radial deflection of particles in free fall along the acoustic axis under gravity around the focal zone due to an interplay of acoustic radiation, gravitational and air drag forces. Our proposed mechanism differs from the acoustic sieve of Li *et al*.^34^ in that millimeter-size particles in air, not in water, can be sorted across a very small volume comprising the focus and that particles in continuous flow can be sorted such that on-the-fly control is facilitated. Hence our approach is serial, while the approach of Li *et al*.^34^ is parallel. Acoustic characterization of the lens and calculation of the trajectories of spherical particles are carried out through finite-element method (FEM) simulations. The mechanism of either size- or material-based particle separation is discussed in detail and contrasted to the characteristics of typical standing SAW microfluidic acoustophoretic particle separation studies.

## Results

**Figure 1:**
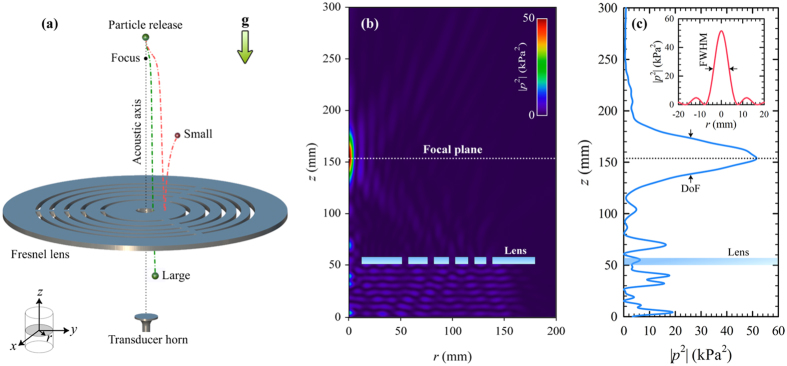
Fresnel lens and its acoustic characteristics. (**a**) Sketch of Fresnel lens and schematic description of size-based particle separation, (**b**) acoustic intensity distribution in 2D over the computational domain at *f*_0_ and *SPL* = 153.0 dB, and (**c**) the variation of intensity over the acoustic axis and the focal plane (inset).

Fresnel lens depicted in Fig. 1(a) is a steel cylindrical disk in air with concentric openings, designed to have a focal length of *f*_*L*_ = 100 mm at *f*_0_ = 25.0 kHz, the resonance frequency of an available Langevin transducer used in levitation experiments^27^. In order to obtain a sharp focus, diffracted acoustic field components emanating from adjacent openings should experience path differences Δ*l*_*i,i*+1_ = *nλ*_0_ where *i* = 1, 2, 3, 4, *n* is an integer and *λ*_0_ = *c*_*a*_/*f*_0_ is the wavelength in air in which speed of sound is denoted by *c*_*a*_. Thus, the radii of concentric holes *r*_*i*_, measured from *r* = 0 to the center of *i*th opening in the radial direction can be quantified as


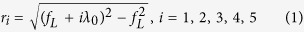


where we assume a path difference of *λ*_0_ between adjacent openings. Fresnel lens thickness is set to *t*_*L*_ = *λ*_0_/2 to facilitate Fabry-Perot resonances across the openings^41^.

Compressible spherical solid particles with diameter *d* are released from the rest above the focus so they fall freely under the influence of the gravitational force **F**_**g**_ = *m***g** along the −*z* direction. When the particles enter the focal zone, they experience the acoustic radiation force **F**_**ac**_ such that they are deflected sideways to a degree dependent on the combined action of **F**_**g**_, **F**_**ac**_ and the air drag force **F**_**d**_ to follow paths depending on their size and acoustic properties. If the acoustic intensity is adjusted properly, particles of the same type with diameters larger than a critical value, i.e. *d* > *d*_*c*_, are deflected less and can be admitted into the central hole, while smaller particles are rejected, as depicted in Fig. 1(a).

Focusing behavior of the lens excited by a plane wave source at *f*_0_ is presented in Fig. 1(b). Source sound pressure level is given by 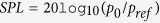 where *p*_0_ is incident pressure field amplitude and *p*_*ref*_ = 20 *μ*Pa. We adopt *SPL* = 153.0 dB (*p*_0_ = 893.4 Pa) to obtain *d*_*c*_ = 0.5 mm for spherical acrylic particles. The lens is capable of sharp focusing, as shown in Fig. 1(b). Maximum *SPL* at the focus is 171.1 dB, thus introducing a significant enhancement of 18.1 dB. In comparison, to trap an acrylic particle with *d* = 1.0 mm against gravity in a levitation experiment with standing acoustic fields, a source *SPL* of at least 165 dB is required. Thus, particle manipulation by the lens not only requires reduced acoustic intensity by more than an order of magnitude, but also offers tunability through adjusting the source *SPL* such that *d*_*c*_ can be adjusted.

In quantifying the focusing characteristics of the Fresnel lens, we consider the squared amplitude of the complex pressure field, i.e. 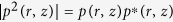, where * denotes complex conjugate, which is proportional to the acoustic intensity. Figure 1(b) shows that intensity is highest at the focus at *r* = 0 on the focal plane marked by the horizontal dotted line, where it decreases abruptly in the radial direction. In contrast, extent of focal spot along the vertical axis is much larger, which indicates that **F**_**ac**_ is much stronger along the radial direction. There exists a weaker side lobe of acoustic intensity in Fig. 1(b), which contributes to the canalization of particles, as will be explained later. From Fig. 1(b), we calculated *f*_*L*_ = 95.6 mm, in good agreement with the design goal of 100 mm.

Analyses of the focusing behavior of the Fresnel lens in Fig. 1 reveal that the most prominent contributions to the focusing comes from the central hole and the two adjacent ring-shaped openings. Thus, one does not have to obtain a perfect plane wave to have the focusing behavior in Fig. 1(b). It is a common practice to excite the first vibrational mode of the Langevin transducer horn in Fig. 1(a), which has azimuthal symmetry so that the ultrasonic wave behaves like a spherical wave in air. Thus, provided that the horn-lens distance is sufficiently high, one can expect approximate plane wave behavior at the lower surface of the lens. The main concern here is the temporal stability of the obtained focal pattern which can be disturbed due to air flow, power fluctuation, horn heating, etc.

Important parameters for specifying the focal spot are the depth of focus (DoF) defined as the distance of the two points on the acoustic axis, at which the acoustic intensity halves, measured from the focus, and the full-width at half maximum (FWHM) along the radial direction. These parameters are presented in Fig. 1(c) which incorporates the plot of acoustic intensity on the acoustic axis and the dotted line in Fig. 1(b). DoF and FWHM calculated from Fig. 1(c) which are 53.0 mm (3.9*λ*_0_) and 7.0 mm (0.5*λ*_0_), respectively, indicate that sub-wavelength focusing of the acoustic field is achieved with the Fresnel lens.

**Figure 2:**
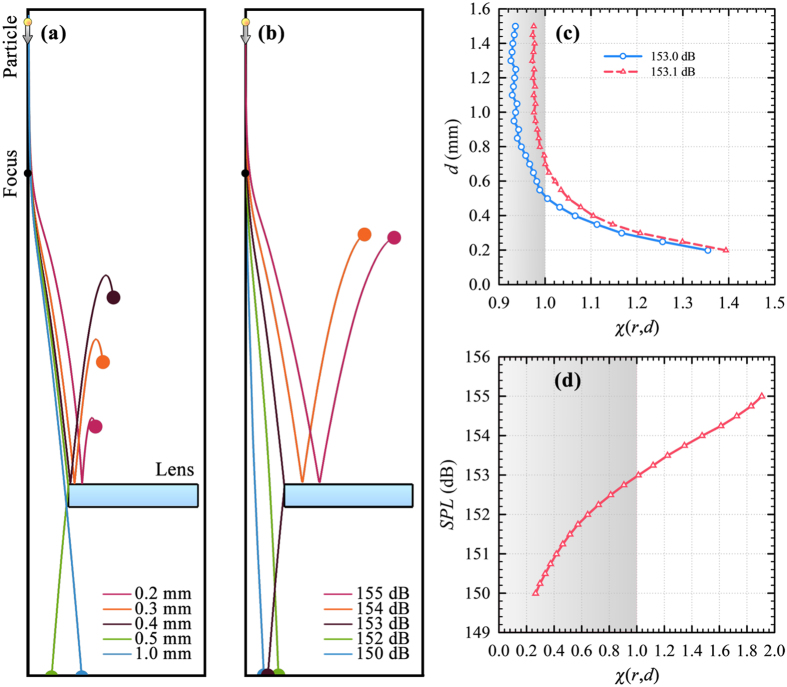
Size-based separation of particles. Calculated trajectories of spherical acrylic particles (**a**) when *SPL* = 153.0 dB for 0.1 mm ≤ *d* ≤ 1.0 mm and (**b**) for *d* = 0.5 mm while *SPL* is varied, as well as the corresponding radial positions of the particles at lens top with respect to (**c**) particle diameter and (**d**) *SPL*. The shaded rectangular areas in (**c**,**d**) denote *χ* ≤ 1 for which particles are admitted.

After obtaining the acoustic field, we calculated the trajectories of spherical acrylic particles released from *h*_*p*_ = 200 mm to free-fall at *f*_0_ and *SPL* = 153.0 dB to demonstrate size-based acoustophoretic separation by means of the lens. Figure 2(a) shows that, depending on their sizes, all particles are deflected radially after they pass through the focal zone. We see that smaller particles are deflected more where particles with *d* = 0.2 mm, 0.3 mm and 0.4 mm bounce off of the top surface of the lens, and thus are not admitted into the central hole, Fig. 2(a). On the contrary, the particles with *d* = 0.5 mm can barely enter the central hole, whereas the ones with *d* = 1.0 mm is safely admitted. Therefore, the lens offers *d*_*c*_ around 0.5 mm at *f*_0_ and *SPL* = 153.0 dB. Although the size-based separation demonstrated in Fig. 2(a) occurs in a serial manner as in the approach of Skotis *et al*.^33^, our approach differs significantly. First, we deal with focused beams to achieve separation, while Skotis *et al*.^33^ utilize a standing field whose nodes are translated by varying relative phases of the two opposing transducers. Furthermore, the particles translate on a horizontal glass plate in the work of Skotis *et al*.^33^ so that contact-free acoustophoresis is not achieved.

Size selectivity of the lens can be tuned by adjusting *SPL*. Figure 2(b) reveals how the trajectory of an acrylic particle with *d* = 0.5 mm depends on *SPL*. Particle is deflected more as *SPL* is increased. As a result, particle can be admitted into the central hole as long as *SPL* ≤ 153 dB. Thus, the lens can also be used for switching where the particles of single species are admitted or not by simply varying *SPL*.

Above observations are quantified in Fig. 2(c,d). The criterion for a particle to pass through the central hole is 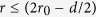 where 2*r*_0_ = 12.0 mm is the radius of central hole. Hence, we define a reduced coordinate 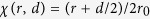. Particles with *χ* ≤ 1 can be safely admitted, while the rest are rejected. Figure 2(c) reveals that *χ* > 1 for particles with *d* < 0.5 mm at *SPL* = 153.0 dB where it drops sharply for larger particles. *d*_*c*_ = 0.52 mm is calculated from Fig. 2(c), in good agreement with the observation in Fig. 2(a). Although *χ* < 1 for particles with *d* > *d*_*c*_, it varies slowly in this range. This is due to the fact that the drag force which acts alone in the radial direction around the lens top does not differ much for the particles with *d* > *d*_*c*_. Slight increase in *SPL* is seen to result in significant variation of *d*_*c*_ in Fig. 2(c). In fact, *d*_*c*_ shifts up to 0.72 mm in response to 0.1 dB increase of *SPL*. Therefore, with an accurate control of source power, one can easily tune *d*_*c*_. Figure 2(d) shows how *χ* increases with *SPL* for an acrylic particle with *d* = 0.5 mm. *χ* varies almost linearly for *SPL* > 153.0 dB where it becomes greater than 1. Thus, the Fresnel lens can also be used for accurate control of radial particle position.

**Figure 3:**
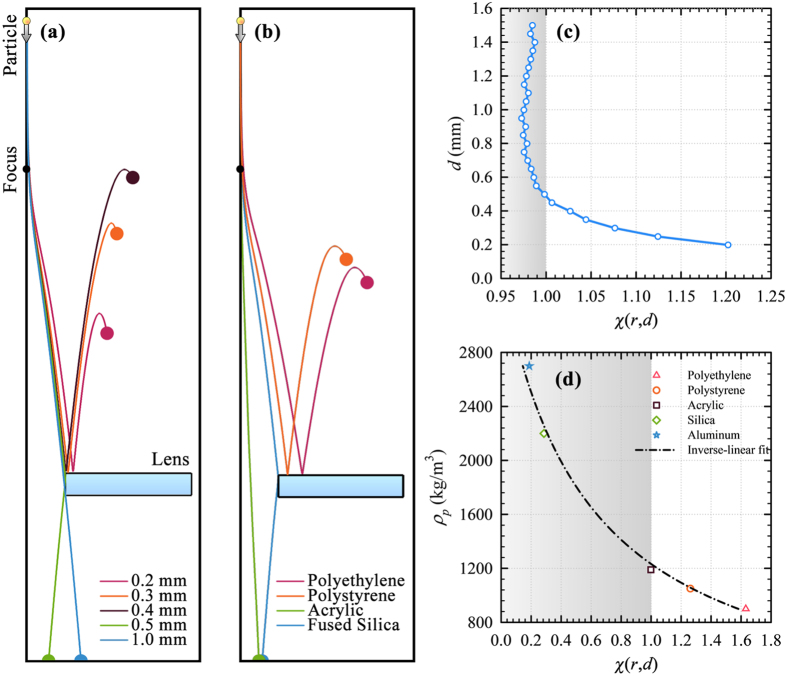
Separation of particles with different material type. Calculated trajectories of spherical aluminum particles (**a**) when *SPL* = 156.7 dB for 0.1 mm ≤ *d* ≤ 1.0 mm and (**b**) for particles with *d* = 0.5 mm made up from different materials when *SPL* = 153.0 dB, as well as the corresponding reduced radial positions of particles at lens top with respect to (**c**) diameter of aluminum particles and (**d**) material density. The shaded rectangular areas in (**c**,**d**) denote *χ* ≤ 1 for which particles are admitted.

Trajectories of spherical aluminum particles calculated for *SPL* = 156.7 dB are presented in Fig. 3(a). 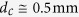 is also obtained for aluminum particles at the specified *SPL*, like acrylic particles in Fig. 2(a). In addition to acrylic and aluminum, Figs 2(a) and 3(a) suggest that the lens can be employed to separate particles with respect to their densities. Thus, particle trajectories for polyethylene, polystyrene and fused silica are calculated when *d* = 0.5 mm and *SPL* = 153.0 dB for consistency. Figure 3(b) shows that fused silica and acrylic particles are admitted into the central hole, while the polystyrene and polyethylene particles, whose densities are close to that of acrylic, are rejected.

Variation of *χ* for aluminum particles with respect to *d* in Fig. 3(c) reveals that *d*_*c*_ = 0.48 mm at *SPL* = 156.7 dB. However, comparison of Fig. 3(c) with Fig. 2(c) reveals that the range of *χ* is much narrower in the case of aluminum due to reduced drag. Figure 3(d) shows that *χ* decreases as the density increases. Curve fitting to the data in Fig. 3(d) with *R*^2^ = 0.99 reveals that *χ* is inversely proportional to particle density, *ρ*_*p*_, with a critical *ρ*_*p*_ = 1230 kg/m^3^ at *SPL* = 153.0 dB.

## Discussion

An acrylic particle with *d* = 1.0 mm weighs 6.1 *μ*N. Thus, magnitude of **F**_**ac**_ that deflects millimeter-size particles should be on the order of micronewtons. Acoustic levitation of such a particle in a standing ultrasonic field at *f*_0_ would require *SPL* of at least 165 dB. Such high intensities can be obtained in air by Langevin-type or magnetostrictive transducers. For instance, levitating a living ladybug without destroying its vitality requires *SPL* = 162 dB which is a typical *SPL* for levitating millimeter-size objects with densities similar to that of water in air^18^. Since we deal with particles with diameters between 0.1 mm and 1.5 mm, we ensure that 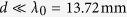, as well as 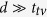 where *t*_*tv*_ is the thermo-viscous boundary layer thickness around the particles which is on the order of 10 *μ*m for such solid particles in air^44^. Hence, **F**_**ac**_, whose origin comes from the nonlinear effect of high-intensity ultrasound^18^, can be calculated as a gradient of scalar potential *U*_*ac*_^45^:


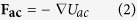


for which *U*_*ac*_ can be written by incorporating second-order perturbation effects in the pressure field *p(r, z*) as^45^


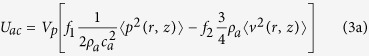



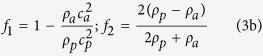


where *v(r, z*) is the speed of oscillating air molecules at the point (*r, z*), *ρ*_*a*_ is the density of air, *V*_*p*_, *ρ*_*p*_, and *c*_*p*_ are the volume, density and longitudinal speed of sound of the particle, respectively. 〈…〉 Denotes time average over a whole period of the acoustic wave. In equation 3, *f*_1_ and *f*_2_ correspond to monopole and dipole coefficients^45^. The monopole coefficient *f*_1_ describes scattering of the acoustic field in a quiescent inviscid fluid from a compressible particle, while the dipole term *f*_2_ corresponds to the displacement of an incompressible particle in the fluid^45^.

If only **F**_**g**_ and **F**_**ac**_ acted, there would be no difference in particle trajectories with respect to size since both forces scale with *d*^3^. What makes a difference is the incorporation of a third force, i.e. **F**_**d**_, which does not scale with *d*^3^. Since particle speed in the radial direction is on the order of 0.1 m/s in our problem, we are dealing with particle Reynolds numbers


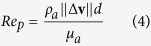


on the order of 10. Standard Stokes drag relation cannot be employed here since it can be used for very small *Re*_*p*_. ||Δ**v**|| in equation 4 is relative speed of particle with respect to background fluid and *μ*_*a*_ = 1.81 × 10^−5^ Pa.s is the dynamic viscosity of air at room temperature. Therefore, we employ the Schiller-Naumann drag model^46^ for the particles which yields much better agreement with experimental data for drag force at such Reynolds numbers:


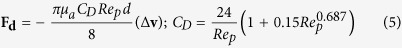


where *C*_*D*_ is the drag coefficient.

**Figure 4:**
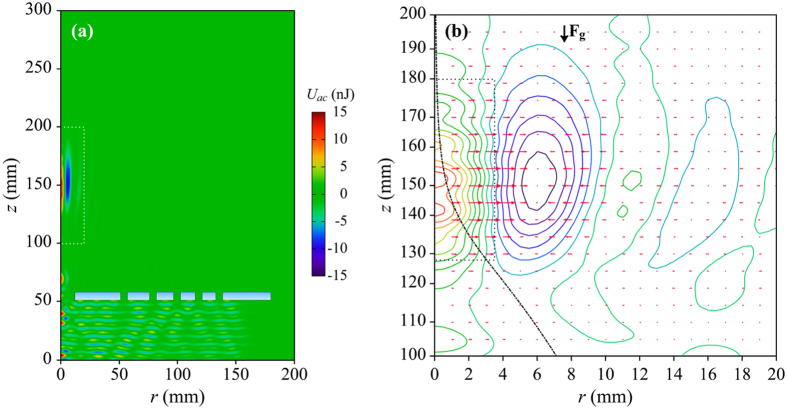
Inspection of acoustic radiation force. (**a**) Distribution of *U*_*ac*_ over the computational domain for a spherical acrylic particle with *d* = 0.5 mm and excitation field at *f*_0_ and *SPL* = 153.0 dB and (**b**) corresponding **F**_**ac**_ vectors in the vicinity of the focus over the dotted rectangle in (**a**) overlaid on the equi-potentials of *U*_*ac*_. Axes in (**b**) are not drawn to scale for clarity, whereas the dash-dotted line in (**b**) represents particle trajectory. The dotted rectangle in (**b**) denotes the region within DoF and HWHM of the focal zone.

We can calculate *U*_*ac*_ by means of *p(r, z*) and *v(r, z*) data obtained from frequency-domain FEM simulation of the acoustic field. Figure 4(a) presents variation of *U*_*ac*_ for a spherical acrylic particle with *d* = 0.5 mm, corresponding to the field distribution in Fig. 1(b). *U*_*ac*_ above the lens is negligible in regions away from the focus where it takes values up to 15 nJ in a narrow region around the focus. On the other hand, *U*_*ac*_ changes sign over the side lobe in Fig. 1(b) such that a plateau along which *U*_*ac*_, and thus its gradient, diminishes appear in between, Fig. 4(a).

Radial and vertical components of **F**_**ac**_ are depicted by the arrow plot in Fig. 4(b). Contours in Fig. 4(b) correspond to the equi-potentials of *U*_*ac*_ to which the **F**_**ac**_ vectors are normal. Direction of **F**_**ac**_ depends on the acoustic contrast factor of the particles, i.e. 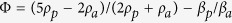, where *β*_*p,a*_ denotes bulk compressibility^45^. If Φ > 0, particles are pushed towards the absolute minimum of *U*_*ac*_, and vice versa. In the realm of microfluidics, biological particles can possess either positive or negative Φ. In contrast, for spherical particles in air with 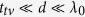, Φ is very close to the ideal value of 5/2 predicted by the Kim model. Thus, in our case, a particle initially in free fall along the acoustic axis can be expected to be deflected sideways by the acoustic radiation force around the focal zone.

When a particle in Fig. 2 or Fig. 3 is released, it first accelerates along the acoustic axis towards the lens due to **F**_**g**_ since *U*_*ac*_ and its gradient are negligible. Since the particle is released slightly offset from the acoustic axis by Δ*r*, breaking the symmetry of *U*_*ac*_ around the particle, and thus a non-vanishing **F**_**ac**_, is ensured. Figure 4(b) shows that **F**_**ac**_ points to the right (increasing *r*) at points within half-width at half maximum (HWHM) of the focus in radial direction, then vanishes, and then reverses direction in the region of the side lobe, with increasing *r*. In contrast, **F**_**ac**_ has negligible *z* component around the focal zone, where its magnitude drops away from the focus in either direction along the acoustic axis. Trajectory of an acrylic particle with *d* = 0.5 mm, denoted by the dash-dotted line in Fig. 4(b) clearly indicates that the particle is pushed more and more radially outward as it passes through the focus. |**F**_**ac**_| the specified particle experiences is as high as 5 *μ*N at HWHM away from the focus in the radial direction. This force is much higher than the gravitational force on the particle, i.e. 0.76 *μ*N. Thus, acceleration of the particle along the radial direction due to **F**_**ac**_ may become as high as 6.5 *g*, where *g* is the gravitational constant.

The fact that smaller particles are deflected more in Fig. 2(a) is in clear contrast to the particle trajectories in microfluidic experiments where solid particles with Φ > 0 deflect more under the influence of standing acoustic field^6^,^7^,^8^,^9^. The discrepancy is due to several factors. First, Stokes drag relation for laminar flow where the drag force scales with *d* can be assumed in the case of microfluidics, while it is more complicated for millimeter-size solid particles in air, as suggested by equation 5. In addition, particles in standing SAW acoustophoresis experience the radiation force continuously in their flow. However, particles in our case feel the radiation force which decays fast in the radial direction so that their radial motion is mostly governed by the drag force away from the focal zone. Thus, radially-inward contribution of the drag force is more pronounced for the larger particles in our case.

**Figure 5:**
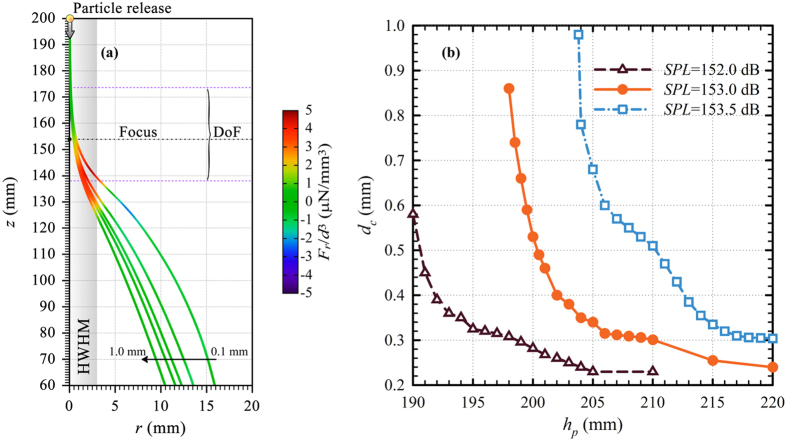
(**a**) A close-up view of the trajectories of spherical acrylic particles with different diameters at *f*_0_ and *SPL* = 153.0 dB along with the normalized radial force and (**b**) variation of *d*_*c*_ as a function of *h*_*p*_ at different source *SPL* values. The axes in (**a**) are not drawn to scale for clarity. *f*_*L*_, DoF and HWHM of the lens are denoted in (**a**) by the horizontal dash-dotted line, dotted lines, and shaded rectangle, respectively.

In order to further investigate the dynamics of particle motion, we present the trajectories of spherical acrylic particles with different diameters at *f*_0_ and *SPL* = 153.0 dB in Fig. 5(a) down to the lens top. The color scale overlaid on the trajectories denotes the net radial force *F*_*r*_ normalized to *d*^3^ which is due to the combined effect of the radial components of **F**_**ac**_ and **F**_**d**_. Above the focus outside the DoF, the particles are not deflected in the vertical direction, while their trajectories are significantly altered within DoF and HWHM in the vertical and radial directions, respectively. It is clearly seen in Fig. 5(a) that particles are first accelerated radially outward within the DoF and HWHM where the primarily radial radiation force dominates, while they start radially decelerating as the radiation force diminishes rapidly. Thus, the rest of the particle trajectories towards the lens top are mainly determined by the gravity in the vertical direction and the opposing drag force which has both radial and vertical components. It is also seen that the variation of the normalized radial force is highest for the smallest particle where the gradient over the particle trajectory diminishes with increasing diameter. That is why particles follow different paths as their sizes differ.

Close inspection of Figs 2(a) and 3(a) reveals that among the particles with *d* < *d*_*c*_, the ones with higher *d* bounce higher off the lens top. If the particles were freely-falling with very low *Re*_*p*_ such that only **F**_**g**_ and opposing **F**_**d**_ in Stokes form acted, their terminal vertical speed (*v*_*z*_) would scale with *d*^2^. Although **F**_**d**_ in equation 5 is more complicated, the same logic holds and larger particles bounce higher. On the other hand, since the contribution of **F**_**d**_ to the vertical acceleration is lower for a denser particle, polystyrene particle in Fig. 3(b) hits the lens surface with higher *v*_*z*_ and bounces higher than the polyethylene bead.

Particle trajectory is a complex function of not only its geometrical and physical attributes and the focused field but also of *h*_*p*_ which determines the particle momentum at the focal zone. Higher *h*_*p*_ leads to higher particle momentum such that the particle spends less time at the focal zone. Since **F**_**ac**_ is essentially radial around the focal zone in Fig. 4(b), only **F**_**g**_ and the vertical component of **F**_**d**_ play an important role in the vertical motion of the particle. Thus, the choice of *h*_*p*_ along with *SPL* is critical in adjusting *d*_*c*_ whose variation with *h*_*p*_ at *SPL* = 152.5 dB, 153.0 dB and 153.5 dB is presented in Fig. 5(b). The plots reveal that *d*_*c*_ increases with *SPL* when *h*_*p*_ is fixed. Furthermore, for a specified *SPL, d*_*c*_ decreases with increasing *h*_*p*_ where the slope increases when the release point is closer to the lens. This is because the particle experiences **F**_**ac**_ for a prolonged period. However, Fig. 1(c) indicates that the vertical component of **F**_**ac**_ is more pronounced for *h*_*p*_ < 190 mm so that particles may be pushed up by the acoustic force. On the other hand, *d*_*c*_ varies slowly when *h*_*p*_ approaches 220 mm. Although this is desirable for a more robust operation, a trade-off should be considered if one demands higher *d*_*c*_ at as small as possible *SPL*. That’s why we choose *h*_*p*_ = 200 mm at *SPL* = 153.0 dB.

Since we utilize a plane-wave source, the system is robust in terms of source-lens distance. Varying the distance does not affect the focusing behavior of the lens. Source position does not affect the trajectories of particles after they pass through the lens since the acoustic intensity below the lens is significantly low in order to alter their paths. Although a plane-wave is an idealization, a good approximation can be realized in the far field of the horn of the transducer. The standing wave pattern observed in Fig. 2(a) due to the adopted source lens distance of 4*λ*_0_ is not capable of trapping particles since the intensity is too low and particles gain very high speeds on the order of 1 m/s.

The robustness of the system can be ensured by stabilizing *SPL* using techniques such as resonance-frequency locking and active feedback of output power. Besides, preventing air flow by conducting experiments in a close vessel also serves to improve system robustness. Moreover, since the radial extent of acrylic particles in Fig. 2(a) is broader than that of aluminum particles in Fig. 3(a) at the lens top, use of less dense particles such as polystyrene and polyethylene could improve system stability in terms of *d*_*c*_.

**F**_**ac**_ is practically unaffected by transiting from acrylic to aluminum in Fig. 3(a) since the terms *f*_1_ and *f*_2_ in equation 3b change negligibly by the increase of *ρ*_*p*_. On the other hand, such a change of material causes a 2.5-fold decrease in **F**_**d**_ in equation 5. Thus, one can expect an increase in the net radially-outward force in the focal zone when the material density is increased. However, while **F**_**g**_ is fixed in the vertical direction, the decrease in **F**_**d**_ results in reduced interaction time with the acoustic force so that one needs to increase *SPL* to compensate. The difference between the two cases is that the discrepancy in aluminum particle trajectories is less pronounced due to the reduction of **F**_**d**_. In fact, irrespective of their size, all aluminum particles are admitted into the central hole at *SPL* = 153.0 dB.

## Methods

Numerical treatment of acoustophoretic particle separation involves two steps: (i) obtaining the steady-state acoustic pressure distribution and (ii) calculating particle trajectories in time under the influence of **F**_**g**_, **F**_**ac**_ and **F**_**d**_. Acoustic-Solid Interaction and the Particle Tracing for Fluid Flow modules of the COMSOL Multiphysics software, which is an implementation of FEM, are employed for the former and latter, respectively. Ignoring the support beams to fix concentric rings of the lens in Fig. 1(a), cylindrical symmetry of the problem can be exploited for the solution of acoustic pressure field in frequency domain. Therefore, through reducing the problem to 2D as in Fig. 1(b), one can obtain complex *p(r, z*), which carries both amplitude and phase information, in cylindirical coordinates where the acoustic axis (i.e. the *z* axis) passing through the central hole is the symmetry axis.

The radius of the central hole is set to 2*r*_0_, while rest of the concentric holes have a width of *r*_0_, where a common length scale denoted by *r*_0_ = 6.0 mm is defined. Total lens radius is set to 180 mm and a plane wave source at *f*_0_ located at *z* = 0 where its radius is *w*_*src*_ = 160 mm is employed. Source-to-lens distance is set to 4*λ*_0_. Computational domain, which is surrounded by absorbing boundaries except the symmetry axis and source, extends along the *r* and *z* axes by 200 mm and 300 mm, respectively. A sketch of the 2D computational domain and the definitions of geometrical parameters can be found in Supplementary Fig. S1.

We used *ρ*_*a*_ = 1.21 kg/m^3^ and *c*_*a*_ = 1.21 m/s for the solution of acoustic wave equation. In addition, Young modulus and Poisson’s ratio of steel for the solution of elastic wave equation are *Y* = 205 GPa and *ν* = 0.28, respectively. A detailed explanation of the formalism adopted in FEM simulations can be found in Supplementary Information. *ρ*_*p*_ of polyethylene, polystyrene, acrylic, fused silica and aluminum for particle-tracing simulations are 900 kg/m^3^, 1050 kg/m^3^, 1190 kg/m^3^, 2200 kg/m^3^, and 2700 kg/m^3^, respectively. Corresponding *c*_*p*_ values are 1950 m/s, 2370 m/s, 2730 m/s, 5970 m/s, and 6420 m/s, respectively.

Time-dependent FEM simulation of particle trajectories involves the release of particles from rest at a point slightly above the focus such that particles do not speed up much in the vertical direction when they encounter the elevated acoustic pressure at the focal zone. Thus, the particles are released from *h*_*p*_ = 180 mm where they are displaced in the radial direction by Δ*r* = 0.05 mm so that their centers do not coincide with the maximum of *U*_*ac*_ at *r* = 0 where the acoustic force is purely vertical due to symmetry. For the calculation of **F**_**ac**_ in equation 2, steady-state *p(r, z*) values calculated in the previous step are employed. Since the particles are expected to spend a few milliseconds in the focal zone, a generalized alpha time-stepping algorithm^47^ with a maximum step size of Δ*t* = 0.5 ms is adopted. Particle coordinates are recorded as a function of time to track whether they pass through the central hole.

## Conclusion

In conclusion, we proposed a method of separating millimeter-size spherical solid particles in air by means of a Fresnel lens through numerical simulations by the finite-element method. Being a thin solid circular disk with a thickness of half wavelength in air, the Fresnel lens is capable of focusing ultrasonic waves at a specified distance with a spot size of 0.5 wavelength FWHM at 25 kHz. This is accomplished by adjusting the distances of the concentric openings of the lens so that an integer multiple of wavelength path difference between the wave components emanating from adjacent openings is obtained at the focus. Concentrating acoustic energy at the focal zone facilitates 18.1 dB enhancement of acoustic intensity.

Due to the fact that the gradient of acoustic intensity is four times larger in the radial direction than in the vertical direction, a particle in free fall moving along the acoustic axis is deflected sideways when it passes through focal zone. The amount of deflection is a function of particle diameter since the acoustic radiation force and the air drag force scale with different orders of the diameter. As a result, a critical diameter of 0.5 mm can be obtained for spherical acrylic particles at a source sound pressure level of 153.0 dB. The critical diameter depends not only on geometrical and physical properties of particles, but also on source sound pressure level and release height. For instance, the critical diameter increases with increasing *SPL*. In addition, particles spend less time in the focal zone when the release height is increased, resulting in a decreased critical diameter at a constant *SPL*. Thus, the critical diameter can reach 1.0 mm when the release height by approximately 5 mm, provided that the release zone is outside the depth of focus of lens. Moreover, density-based separation of particles with identical geometry is also demonstrated. It is shown that the axial position of particles at lens top is inversely proportional to material density, and thus particles with similar densities can be separated by appropriately setting the release height and *SPL*.

## Additional Information

**How to cite this article:** Cicek, A. *et al*. Acoustophoretic separation of airborne millimeter-size particles by a Fresnel lens. *Sci. Rep.* **7**, 43374; doi: 10.1038/srep43374 (2017).

**Publisher's note:** Springer Nature remains neutral with regard to jurisdictional claims in published maps and institutional affiliations.

## Supplementary Material

Supplementary Information
