# Supplementary material for: Acoustophoretic separation of airborne millimeter-size particles by a Fresnel lens
Source: Sci Rep. 2017 Mar 2;7:43374. doi: 10.1038/srep43374 (PMC5333108; doi:10.1038/srep43374)
Supplement: Supplementary Information [file srep43374-s1.pdf]

Acoustophoretic separation of airborne millimeter-size particles by a Fresnel lens

Ahmet Cicek[1,*], Nurettin Korozlu[1], Olgun Adem Kaya[2], and Bulent Ulug[3]

[1]Department of Nanoscience and Nanotechnology, Faculty of Arts and Science, Mehmet Akif Ersoy University, 15030 Burdur/Turkey.
[2]Department of Computer Education and Educational Technology, Faculty of Education, Inonu University, 44280 Malatya/Turkey.
[3]Department of Physics, Faculty of Science, Akdeniz University, 07058 Antalya/Turkey.
[*]ahmetcicek@mehmetakif.edu.tr

ABSTRACT

Supplementary information

Obtaining $p(r,z)$ in FEM simulations involves simultaneous solution of the acoustic wave[1,2] and the elastic wave[2,3] equations:

$$\nabla\cdot\left(\frac{1}{\rho_a}\nabla p(r,z)\right)+\frac{\omega^2 p(r,z)}{\rho_a c_a^2}=0 \tag{1}$$

$$\nabla\cdot\sigma=-\rho_s\omega^2\mathbf{u} \tag{2}$$

in the air and solid regions of the computational domain, respectively. In equation 1 and equation 2, ρ_a and c_a are the density of and the speed of sound in air while σ and $\mathbf{u}$ are the Cauchy stress tensor and the displacement of atoms in the solid, respectively, where $\omega = 2\pi f_0$ is the angular frequency. Proper boundary conditions at the air-solid interfaces can be written as[2]

$$\mathbf{n_a}\cdot\left(\frac{1}{\rho_a}\nabla p(r,z)\right)=a_n;\ \ \texttt{on}\ \ \Sigma_a \tag{3a}$$

$$\mathbf{f_s}=-\mathbf{n_s}p(r,z);\ \ \texttt{on}\ \ \Sigma_s \tag{3b}$$

Σ_a and Σ_s in equation 3a and equation 3b denote the air-solid boundary when looked from the air and solid region, whereas $\mathbf{n_a}$ and $\mathbf{n_s}$ stand for the corresponding boundary normals, respectively. Briefly, equation 3a tells us that normal acceleration due to the displacement of solid atoms is responsible for the oscillations of air molecules, thus being named as the normal acceleration boundary condition[2]. In return, pressure variations in the air region induce a normal load denoted by $\mathbf{f}_s$ in equation 3b on the solid part of the problem[2].

Supplementary Figure S1 presents a depiction of the 2D computational domain exploiting axial symmetry and definition of relevant geometrical parameters:

An adaptive meshing scheme with triangular elements is adopted. The maximum element size is set to $t_L/5$ in the lens region, whereas the rest of the computational domain is meshed with a maximum element size of 1.0 mm. However, considering the expected trajectories and the interaction time scale of the particles, a maximum element size of 0.5 mm around the symmetry axis is adopted. Under these conditions, computational domain is meshed with 180050 elements in total, where the minimum and average element qualities are 0.65 and 0.98, respectively. Besides, since the particles are expected to spend a few milliseconds in the focal zone, a generalized alpha time-stepping algorithm[4] with a maximum step size of $\Delta t = 0.5$ ms is adopted. Particle coordinates are recorded as a function of time to track whether they pass through the central hole.

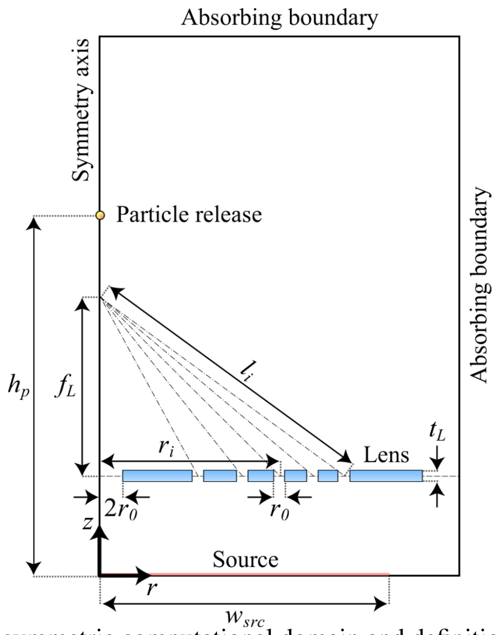

Figure S1. 2D axially-symmetric computational domain and definitions of geometrical parameters.

References

1. Miyashita, T. Sonic crystals and sonic wave-guides. *Measurement Science and Technology* **16**, R47 (2005).
2. Yoon, G. H., Jensen, J. S. & Sigmund, O. Topology optimization for acoustic-structure interaction problems. In *IUTAM Symposium on Topological Design Optimization of Structures, Machines and Materials*, 355–364 (Springer, 2006).
3. Vasseur, J. *et al.* Phononic crystal with low filling fraction and absolute acoustic band gap in the audible frequency range: A theoretical and experimental study. *Physical Review E* **65**, 056608 (2002).
4. Hulbert, G. M. & Jang, I. Automatic time step control algorithms for structural dynamics. *Computer Methods in Applied Mechanics and Engineering* **126**, 155–178 (1995).
